# Supplementary material for: Reservoir of Antibiotic Residues and Resistant Coagulase Negative Staphylococci in a Healthy Population in the Greater Accra Region, Ghana
Source: Antibiotics (Basel). 2022 Jan 17;11(1):119. doi: 10.3390/antibiotics11010119 (PMC8772731; doi:10.3390/antibiotics11010119)
Supplement: Supplementary file 1 [file antibiotics-11-00119-s001.zip › antibiotics-1532595-supplementary.pdf]

Supplementary Materials

Reservoir of Antibiotic Residues and Resistant Coagulase Negative Staphylococci in a Healthy Population in the Greater Accra Region, Ghana

Samuel Oppong Bekoe *, Sophie Hane-Weijman, Sofie Louise Trads, Emmanuel Orman, Japheth Opintan, Martin Hansen, Niels Frimodt-Møller and Bjarne Styrishave

Table S1. Validation parameters summarised for the investigated antibiotics.

	**AMX**	**AMP**	**CFX**	**CPF**	**ETM**	**MTZ**	**STX**	**TCC**	**TMP**
**LDR (ng mL^{-1})**	500 to 5000 ng mL^{-1} for all analytes								
**r^2**	0.941	0.991	0.983	0.989	0.987	0.977	0.993	0.948	0.984
**Instrument LOD (ng mL^{-1})**	1.18×10^3	5.57×10^2	1.18×10^3	5.50×10^2	4.61×10^2	1.11×10^3	3.72×10^2	9.25×10^2	3.10×10^2
**Instrument LOQ (ng mL^{-1})**	3.57×10^3	1.69×10^3	3.57×10^3	1.67×10^3	1.40×10^3	3.37×10^3	1.13×10^3	2.80×10^3	9.40×10^2
**Instrument precision (CV)**	17.0	6.1	11.2	5.7	4.1	8.7	4.0	8.0	3.2
**Accuracy ± CV**	111 ± 7	107 ± 5	99 ± 3	101 ± 8	108 ± 12	112 ± 7	101 ± 9	104 ± 3	92 ± 11

Linear dynamic range (LDR), coefficient of correlation (r^2), instrument LOD and LOQ were determined from linear regression curves. Concentration units of parameters investigated are in ng mL^{-1}. AMX – Amoxicillin, CFX – Cefuroxime, ETM – Erythromycin, MTZ – Metronidazole, STX – Sulphamethoxazole, TCC – Tetracycline and TMP – Trimethoprim

Table S2. Overview of samples collected and subsequent isolation of coagulase-negative strains of staphylococcus

Collection site	Total samples collected (% of total samples)	Number of urine samples with CoNS (% of total urine samples)	Number of isolates (% of total CoNS) *
Dodowa	201 (50.1)	30 (14.9)	37 (58.7)
Korle-Gonno	200 (49.9)	17 (8.5)	26 (41.3)
Total	401	47(11.7)	63

**Some samples contained multiple strains of CoNS.*

Table S3. Overview of Results from the Detection of Antibiotic Residues

Presence of Antibiotics	Samples containing CoNS	Samples without CoNS (% of total without CoNS)	Total Number of Samples (% of total for both types of samples)
Yes	42 (89.4)	47 (13.3)	89 (22.2)
No	5 (10.6)	307 (86.7)	312 (77.8)
Total	47	354	401

Table S4. Classification of samples with detected antibiotics based on location

Location	Samples containing CoNS (% of total with CoNS)	Samples without CoNS (% of total without CoNS)	Total Number of Samples (% of total for both types of samples)
Dodowa	28 (52.8)	25 (47.1)	53 (59.6)
Korle-Gonno	14 (38.9)	22 (61.1)	36 (40.4)
Total	42	47	89

Table S5. Frequency of antibiotic residues detected in the urine samples

Antibiotic	Dodowa			Korle-Gonno			Total number of antibiotics in the 89 samples (A + B)
	Samples containing CoNS	Samples without CoNS	Sub-total of samples containing antibiotic (A)	Samples containing CoNS	Samples without CoNS	Sub-total of samples containing antibiotic (B)	
Amoxicillin	1	0	1	2	4	6	7
Ampicillin	2	4	6	1	1	2	8
Ciprofloxacin	20	3	23	12	13	25	48
Metronidazole	2	0	2	1	1	2	4
Sulphamethoxazole	4	1	5	0	2	2	7
Tetracycline	17	23	40	6	10	16	56
Trimethoprim	4	1	5	3	2	5	10
Total	50	32	82	25	33	58	140

File S1: Details on the conduct of survey

A. INFORMATION TO THE PARTICIPANT ABOUT THE PROJECT

The Antibiotic Drug Use, Monitoring and Evaluation of Resistance Project is a research-building collaboration between Ghana and Denmark. It is funded by DANIDA (Danish International Development Assistance). The project strives to increase knowledge on antibiotic consumption, use and resistance, and further to spread this knowledge in order to gain awareness on the importance of prudent antibiotic use principles. The aim of the specific study that you are being asked to participate in is to investigate if there are small traces of pharmaceuticals in the bodies of healthy, fit members of the community, such as yourself. We strive to gather about 200 samples from your community, with a wide range of ages and both male and female. You have been selected randomly, without bias, based on your healthy, fit appearance. Participation is 100% voluntary, and you are in no way obligated to participate. If you choose to participate, the researcher will:

1. Explain the project to you (by sharing this information with you).
2. Provide you with a bottle of drinking water for you to consume during the questionnaire, to make the collection of urine easier.
3. Administer the questionnaire, containing simple questions about your health, education, diet and water consumption.
4. Hand you a sterile container for gathering your urine sample. The sample will be labeled with a random code to protect your privacy.
5. Store the sample in an ice chest and transport it to the nearest lab where the initial tests will be conducted the same day.
6. Thank you for your participation and be on his or her way.

Because you are considered a fit and healthy individual, it is very unlikely that any bacteria will be found in your urine. However, in the unlikely case that bacterial growth is found, we will inform you within a week and refer you to a doctor for paid further tests and, if necessary, medical treatment.

Thank you for your participation!

B. CERTIFICATE OF CONSENT

I have been invited to participate in a research project about health seeking behaviour and medicine use. I understand that every one of the 200 participants in the Dodowa and Korle-Gonno area in this study was selected randomly and without bias and that my urine sample will be collected, around 80 ml -150 ml, and used for a large, international study to improve knowledge on pharmaceutical use, its sources and resistance as well as provide scientific data to inform policy. I understand that my urine sample will be screened for bacteria and pharmaceuticals and that I will be provided feedback after the initial bacterial screening within 4 to 5 working days by phone, to be informed of the presence of bacteria in my urine and, if necessary, provided a voucher to seek medical attention in the unlikely event that a bacterial infection is found. I understand that my urine will not be used for any other purpose and disposed after 9 months upon completion of the study. I understand that all information from the questionnaire as well as urine sample results will be kept completely confidential and the only use of my name will be to provide feedback to me, and that a random code will be assigned to my sample to ensure my privacy. I have read the foregoing information, or it has been read to me. I have had the opportunity to ask questions about it and any questions.

Any questions or concerns I may have can be answered by contacting either Dr. Opintan, at +233302665404 or Prof. Newman at +233244329266, who are both from the University of Ghana Medical School, at the Department of Microbiology. Any questions I have, have been answered to my satisfaction. I consent voluntarily to be a participant in this study.

Print Name of Participant: ____

Signature / Thumbprint of Participant: _______________or [ ]

Date: ______________________

Day / month / year

If Participant is a minor,

Signature / Thumbprint of Guardian: ____________________or

For Participants who are illiterates[1]

I have witnessed the accurate reading of the consent form to the potential participant, and the individual has had opportunity to ask questions. I confirm that the individual has given consent freely.

Name of Witness: ________________________________

Signature / Thumbprint of Witness: ___________________or

Date: ___________________________

Day / month / year

[1] A literate witness must sign (if possible, this person should be selected by the participant and should have no connection to the research team). Participants who are illiterate should include their thumb print as well.

C. INFORMED CONSENT PROCEDURE

INTRODUCTION

Interview Serial/Sample Number: ________________________

(should be the same as label on sample)

Before beginning interview, please ensure that participant appears to be healthy. Ask if they have taken any medication and attempt to identify using catalogue. If you suspect they have taken antibiotics, please explain to them that they cannot be included in study and move on to next possible participant.

If participant is excluded, please note their name and the reason why they were excluded, to enable a total count of participants excluded and sampled.

Section 1 – Demographics

Study site	Korle-Gonno ☐ Dodowa ☐
Name of community where interviewee resides	____
Age of interviewee	__________________ years
Gender	Male ☐ Female ☐
Marital Status	Married or living together☐ Divorced/separated ☐<br>Widowed ☐ Never married/never lived together ☐
Highest education level attained by interviewee	No education/Illiterate ☐ Primary level ☐ Middle/JSS/JHS ☐<br>Secondary/SSS/SHS/Vocational/Technical ☐ Higher ☐<br>Don't Know ☐
Occupation	______________________________________
Health Insurance status of interviewee	NHIS ☐ Other insurance ☐<br>None ☐ Don't know ☐
Validity of Health Insurance	Valid ☐ Not valid ☐<br>Not Applicable ☐ Don't know ☐
Date of Interview	_______/______/_______
Name of interviewer	______________________________________

Language of interview	English ☐ Akan ☐ Ga ☐ Ewe ☐ Nzema ☐ Dagbani ☐ Hausa ☐ Other ☐ specify __________
Language of respondent	English ☐ Akan ☐ Ga ☐ Ewe ☐ Nzema ☐ Dagbani ☐ Hausa ☐ Other ☐ specify __________
Translator used	Yes ☐ No ☐

Section 2 - Medicine

1. How is your health today? Well ☐ Unwell ☐

   ____

   (If participant suggest he/she is unwell, continue to question 2, otherwise move to question 3)

2. So how are you managing your ill-health?

   ____

3. Are there any medicines that you use either regularly or once a while (other than antibiotics)?

   ____

   ____

   (probe for both orthodox and traditional medicines)

4. Have you taken any medication within the past 14 days? Yes ☐ No ☐
5. If, yes – what medication? (Show catalogue if necessary)

   ____

   ____

6. How do you take the medicines (probe for drug administration information)?

   ____

   ____

7. For what conditions are you using the medicines to manage (probe for each medicine stated)?

____

____

8. How did you know that this medicine(s) is indicated for your condition? (if medicines are self-prescribed)

____

____

Note: questions 5 – 8 applies to all medicines stated in questions 3 and 4

Section 3 - Diet

1. Do you consume animal or fish products in your diet? Yes ☐ No ☐

   (If not skip to question 4).

2. What type of animal or fish products do you consume? (tick as many as apply)

   Chicken ☐ Beef ☐ Goat/Sheep meat ☐

   Pig meat ☐ Bush meat ☐ Snails ☐

   Eggs ☐ Fish ☐ Other ☐ (Specify) ____________________

3. Which animal or fish products did you consume today and yesterday? (Tick as many as apply)

   Chicken ☐ Beef ☐ Goat/Sheep meat ☐

   Pig meat ☐ Bush meat ☐ Snails ☐

   Eggs ☐ Fish ☐ Other ☐ (Specify) ____________________

4. What is your main drinking water source?

   Bottled water ☐ Pure water (Sachet water) ☐ Tap water ☐

   Pond/River water ☐ Other ☐ (Specify) ____________________

5. Home Filtered Yes ☐ No ☐

   Boiled water Yes ☐ No ☐

6. Which drinking water sources have you used today and yesterday? (Tick as many as apply)

   Bottled water ☐ Pure water (Sachet water) ☐ Tap water ☐

Pond/River water ☐ Other ☐ (Specify) ____________________

7. Is there anything else you wish to share with me or questions you may have?

____

____

8. Can I please have you telephone contact in case I need to call you to seek more information or clarification?

   Tel. No____

Notes from field.

**Remedy Used**	**Date used***	**Dosage (dose / Frequency /duration)**	**Reason for use**	**Prescriber (e.g. Dr; nurse, pharmacist; self; relative; peddler; drug store attendant etc)**	**Source of Remedy (Peddler/ hospital/ drug store**	**Treatment Outcome**

*Date Used: Is exact/rough date available, please state otherwise indicate regularity of use e.g., 10 capsules weekly or monthly or when necessary/when symptoms appear etc.

Table S6. Residual Concentrations of the Antibiotics in the Urine Samples

Analyte	No. of samples containing quantifiable levels (N = 75)	Median (ng mL^{-1})	Range (Min - Max) (ng mL^{-1})
Amoxicillin	7	10.3	1.89 – 17000
Ampicillin	5	5.4	3.52 – 1180
Ciprofloxacin	48	14.55	1.7 – 1460
Metronidazole	3	167	11.9 – 4000
Sulphamethoxazole	7	16.4	5.19 – 154
Tetracycline	41	26.7	4.19 – 986
Trimethoprim	9	2.91	1.44 – 5.41

Table S7.EUCAST Susceptibility/Resistance breakpoint data (EUCAST, 2020)

Antibiotic	Zone diameter breakpoint (mm)		Antibiotic	Zone diameter breakpoint (mm)	
	S ≥	R <		S ≥	R <
Penicillin V	26	26	Chloramphenicol	18	18
Cefoxitin	25	25	Fusidic acid	24	24
Gentamicin	22	22	Sulphamethoxazole*	16	11
Erythromycin	21	18	Trimethoprim	17	14
Clindamycin	22	19	Rifampicin	26	23
Tetracycline	22	19	Novobiocin	16	16
MIC breakpoint (mg L^{-1}) for E-test					
Vancomycin	4	4			

**Value from Department of Clinical Microbiology, Hvidovre Hospital (DK).*

Table S8. Mass spectrometry specific parameters in the validated LC-MS/MS analytical method indicating ion transitions for the test antibiotics and 3 deuterated internal standards.

	**Retention time (mins)**	**Mode of Ionization**	**Precursor ion**	**Quantifier > Qualifier**	**Dwell Time (MS)**	**DP (V)**	**FP (V)**	**EP (V)**	**CE (eV)**	**CXP (V)**
Amoxicillin	1.98	Negative	364.3	222.8 > 302.8	200	-50	-200	-10	-20	-10
Metronidazole	14.46	Positive	172	128.0 > 82	200	25	80	7	20/37	19/7
Trimethoprim	16.07	Positive	291	230 > 261	100	40	140	6	34/36	15/19
d3-Trimethoprim	-	Positive	294	230 > 264	200	40	140	6	34/36	15/19
Ciprofloxacin	17.36	Positive	332	314 > 288.3	200	55	175	10	30	20
d8-Ciprofloxacin	-	Positive	340	322.3 > 296	200	55	175	10	30	20
Tetracycline	19.38	Negative	443.2	357.9 > 186.9	200	-20	-200	-10	-30	-10
Ampicillin	22.93	Negative	348	270 > 215	100	-20	-95	-8	-20/-35	-4/-12
Cefuroxime	24.19	Negative	423.2	317.8 > 206.7	200	-20	-100	-10	-10	-15
Sulphamethoxazole	23.34	Negative	252	156 > 92	200	-30	-110	-5	-20/-36	-7
d4-Sulphamethoxazole	-	Negative	256	159.7 > 96	200	-30	-110	-5	-20/-36	-7
Erythromycin	24.89	Positive	734.2	158.3 > 576.3	200	20	100	10	35	30

KEY: DP – Declustering Potential**; FP –** Focussing Potential**; EP –** Entrance Potential**; CE –** Collision Energy**; CXP –** Collision Cell Exit Potential.
